# A Case Report on Rhabdomyolysis After Multiple Bee Stings

**DOI:** 10.7759/cureus.9501

**Published:** 2020-07-31

**Authors:** Krishna Constantino, Alec J Pawlukiewicz, Leigh Spear

**Affiliations:** 1 Emergency Medicine, Brooke Army Medical Center, Fort Sam Houston, USA

**Keywords:** rhabdomyolysis, bee stings, envenoming syndrome, acute kidney injury, envenomation

## Abstract

Envenoming syndrome is an uncommon condition associated with significant morbidity and mortality following multiple Hymenoptera stings. We review the case of a 90-year-old male who presented after receiving greater than 100 bee stings and was found to have rhabdomyolysis with concomitant acute kidney injury. Physicians should consider envenoming syndrome in all patients presenting with greater than 50 bee stings, despite hemodynamically stability upon initial presentation.

## Introduction

The order Hymenoptera notably includes three families of stinging insects: Apidae (bees), Vespidae (wasps and hornets), and Formicidae (ants) [1]. Hymenoptera stings typically result in localized erythema, edema, and pain [2]. Prior sensitization to the venom results in immediate hypersensitivity reactions, such as angioedema and anaphylaxis, and can occur with a single sting [1]. Associated with multiple stings are rarer complications, such as myocardial infarction, acute kidney injury (AKI), rhabdomyolysis, thrombocytopenia, hepatic injury, seizures, and acute cerebral infarction [2-4]. We present a case of a 90-year-old male who arrived to the emergency department several hours following hundreds of bee stings with facial angioedema. He was discovered to have AKI and rhabdomyolysis.

## Case presentation

A 90-year-old male presented to the emergency department with a chief complaint of facial swelling after having been stung by more than 50 bees the day prior. The patient was unable to remove the stingers on his own. While the patient initially reported blurry vision, this symptom was likely due to bilateral eyelid edema as complete resolution was noted after manual eyelid opening. He denied throat tightness, difficulty breathing, voice changes, nausea, vomiting, or diarrhea. Additionally, the patient denied lightheadedness, recent falls, recent trauma, or strenuous exertion. Initial vital signs at time of presentation included blood pressure 151/74 mm Hg, heart rate 61 beats per minute, respiratory rate 16 breaths per minute, oxygen saturation 98% on room air, and temperature 98.7 degrees Fahrenheit. The patient’s physical exam was significant for dozens of stingers present on his face, scalp, bilateral upper extremities and lower extremities, and chest. He had angioedema of bilateral ears, bilateral periorbital areas, upper and lower lip without involvement of the tongue, soft palate, or uvula. The remainder of the physical exam was without significant findings. The patient had no clinical evidence of anaphylaxis, and epinephrine was not administered. The patient was treated with 25 mg of intravenous (IV) diphenhydramine, 125 mg of IV methylprednisolone, and 20 mg of IV famotidine. The patient’s laboratory results were significant for a leukocytosis of 20,270 cells/µL and a creatinine of 1.86 mg/dL (0.67-1.17 mg/dL) with an unknown baseline renal function. He was admitted for observation due to impaired functional status secondary to the periorbital edema. Shortly after admission, the patient was noted to have dark, cola-colored urine, and a urinalysis and creatine kinase (CK) were obtained. Urinalysis was significant for 3+ blood and five red blood cells/high-power field and his initial CK level was 7,968 international units (IU)/L (24-170 IU/L), and IV crystalloid infusion was initiated. His subsequent CK level drawn eight hours later demonstrated an increase to 13,246 IU/L and an isotonic bicarbonate infusion was started to treat his rhabdomyolysis. His CK levels peaked three days after his bee attack with improvement of his creatinine and resolution of his facial angioedema by the time of hospital discharge.

## Discussion

Bee stings can have various clinical presentations, largely dependent on the number of stings, victim size, and individual characteristics, e.g. comorbidities, immune status, and prior sensitization [5-7]. The most common presentation is a localized reaction and is typically seen in individuals with few stings and without hypersensitization to the venom. Individuals may also present with allergic systemic symptoms ranging from mild urticarial reactions to anaphylaxis after only a single sting [8]. A third presentation associated with multiple bee stings is massive envenomation that results in systemic toxicity with multi-organ failure. 

Massive envenomation, defined as a minimum of 50 stings, causes systemic toxicity due to the amount of venom inoculated, with an estimated lethal dose of 500-1,000 stings [5,7,9]. Bee venom is a complex mixture of amines and peptides, most notably mellitin, the main and most toxic compound and phospholipase A2, the most immunogenic peptide [2,7]. In addition to causing the pain associated with bee stings, mellitin functions as a lytic peptide that destroys cells and leads to intravascular hemolysis [7,10]. Manifestations of envenoming syndrome include rhabdomyolysis, AKI, myocardial necrosis and infarction, disseminated intravascular coagulation, seizures, stroke, thrombocytopenia, and centrilobular necrosis of the liver [2,6,10,11]. Mortality from massive envenomation is often due to cardiac arrest or renal failure [11,12].

The mechanism behind the development of rhabdomyolysis in massive envenomation is not clearly understood. It has been suggested that mellitin is the main factor involved due to its ability to insert pores in cell membranes, causing hydrolysis and cell death [6,7]. Experimental studies with bee venom show increases in CK with tubular myoglobin deposition, and microscopic examination of muscle tissue exposed to bee venom shows characteristics of rhabdomyonecrosis associated with diffuse inflammation and vascular congestion [2,6]. In envenoming syndrome-induced rhabdomyolysis, AKI results from a combination of pigment nephropathy and direct cytotoxic effects of the venom on renal tubules [2,6,11]. Patients who develop AKI secondary to envenoming syndrome have mortality rates as high as 22% [2,12]. 

The diagnosis of envenoming syndrome can be difficult upon initial presentation as systemic toxicity is often delayed resulting in premature discharge. Initial symptoms of toxicity are often non-specific and may include weakness, myalgias, nausea, vomiting, and diarrhea which then evolve into hemolysis, hematuria, and AKI. Hematuria typically occurs within 24 hours of the envenoming incident followed by elevation of CK levels and clinical evidence of kidney injury within the next 48 hours [5,6]. Several case reports exist that describe patients being discharged after having been evaluated for multiple bee stings only to return hours later with symptoms consistent with envenoming syndrome requiring further intervention [5,12].

## Conclusions

Envenoming syndrome is an uncommon condition that carries significant morbidity and mortality. Initial presentation may be benign resulting in a delay of necessary medical care. Emergency providers should keep a high clinical suspicion for this diagnosis in all patients presenting with greater than 50 Hymenoptera stings especially in children and the elderly due to a smaller volume of distribution. Furthermore, providers should have a low threshold for laboratory testing and prolonged observation periods in patients presenting with multiple bee stings even in the absence of early symptoms.
